# Iron (II) Citrate Complex as a Food Supplement: Synthesis, Characterization and Complex Stability

**DOI:** 10.3390/nu10111647

**Published:** 2018-11-03

**Authors:** Daniele Naviglio, Maria Michela Salvatore, Marianna Limatola, Ciro Langella, Stefano Faralli, Martina Ciaravolo, Anna Andolfi, Francesco Salvatore, Monica Gallo

**Affiliations:** 1 Department of Chemical Sciences, University of Naples Federico II, via Cintia, 4, 80126 Naples, Italy; naviglio@unina.it (D.N.); mariamichela.salvatore@unina.it (M.M.S.); limatolamarianna@gmail.com(M.L.); martinaciaravolo@gmail.com (M.C.); andolfi@unina.it (A.A.); frsalvat@unina.it (F.S.); 2 Department of Molecular Medicine and Medical Biotechnology, University of Naples Federico II, via Pansini, 5, 80131 Naples, Italy; cirolangella88@gmail.com; 3 Medical Center, Piazzale Luigi Cadorna, 9, 20123 Milan, Italy; info@faralli.net

**Keywords:** iron citrate (II), food supplement, nutraceuticals, iron deficiency, anaemia

## Abstract

Iron deficiency represents a widespread problem for a large part of the population, especially for women, and has received increasing attention in food/supplement research. The contraindications of the iron supplements commercially available (e.g., imbalances in the levels of other essential nutrients, low bioavailability, etc.) led us to search for a possible alternative. In the present work, a rapid and easy method to synthetize a solid iron (II) citrate complex from iron filings and citric acid was developed to serve, eventually, as a food supplement or additive. In order to state its atomic composition and purity, an assortment of analytical techniques was employed (e.g., combustion analysis, thermogravimetry, X-ray diffractometry, UV/Vis spectrophotometry, etc.). Results demonstrate that the synthesized crystalline solid corresponds to the formula FeC_6_H_6_O_7_∙H_2_O and, by consequence, contains exclusively iron (II), which is an advantage with respect to existing commercial products, because iron (II) is better absorbed than iron (III) (high bioavailability of iron).

## 1. Introduction

Iron is a trace element necessary for the human body because it enters the constitution of haemoglobin, myoglobin and different iron-dependent enzymes [1]. In addition to erythropoiesis, iron is essential for mitochondrial function, DNA synthesis and repair, and many enzymatic reactions required for cell survival. Iron deficiency (generally indicated as anaemia) may contribute to cognitive developmental defects in children, poor physical performance, and unfavourable pregnancy outcomes [2,3,4,5,6]. Dysregulation of iron metabolism contributes to various human pathologies, including iron overload diseases and cancer. Great strides in understanding iron metabolism have been made in the past several years, including the identification of new essential proteins in human iron metabolism and novel roles for iron in both normal cellular processes and in pathologies such as cancer [7]. In addition, iron is deeply linked to cell death; traditionally, iron is thought to contribute to cell death pathways through reactive oxygen species (ROS) production. Iron and ROS are increasingly recognized as important initiators and mediators of cell death in a variety of organisms and pathological situations. Recently, an ROS-independent role for iron in modulating apoptosis (programmed cell death) was defined. Furthermore, an entirely new mode of iron-dependent cell death, termed ferroptosis, was described [8]. The absorption of iron essentially depends on three main factors: (1) the iron content of the diet; (2) the liberation of iron contained in food by the digestive processes and its availability to be taken from the mucosa of the small intestine; (3) intestinal conditions at the intraluminal level that greatly influence the final availability of iron. Moreover, there are two forms of iron: iron-heme and non-heme iron (the latter is also called inorganic iron), because their bioavailability is very different and greatly affects the performanceof the potential absorption of iron in the diet [9].

Heme iron is found only in foods of animal origin, particularly in meat, as it is present in muscle haemoproteins (dairy products, on the contrary, are completely devoid of them), and also in haemoproteins of fish and eggs, and it is absorbed better than non-heme iron from foods. Its absorption, which is around 25% (*w*/*w*), is independent of the composition of the diet because it is absorbed intact as a porphyrin complex without interference from the other constituents of the diet [10]. The absorption of non-heme iron, on the contrary, depends on the composition of the diet and the presence of enhancers and inhibitors of absorption [11] as well as the oxidation state of the metal [12]. Non-heme iron is found in cereals and vegetables and its absorption is generally low; only about 2% to 20% of the iron available from vegetable sources is absorbed. The absorption of the iron by the organism takes place mainly in the duodenum and in the proximal section of the jejunum, and transited through the enterocyte to release the free iron into blood circulation. Therefore, ferrous iron, being much more soluble than ferric iron (at the same pH value), can be more easily absorbed, providing a greater opportunity for chelation and a higher exposure of the mucosa to solubilized forms before precipitation occurs [13]. The chelate forms of ferric iron that remain in solution can probably be taken from the mucosal cells; in any case, the iron made free is transferred to the plasma in a form that can be linked to the transferrin. Ferric iron is absorbed only after its reduction to ferrous iron; this reduction process can occur in the stomach [14]. The indications given for dietary iron intake are based on the losses and quantities necessary for growth and to build up reserves but taking into account the bioavailability of this nutrient in the diet itself. Iron deficiency is responsible for anaemia, a widespread disease in Europe [15]. Clinically, iron deficiency anaemia occurs with asthenia, pallor, tachypnoea and tachycardia [16]. Anaemia should be identified on the basis of haemoglobin values: values between 13 and 16 g/dL in men and between 12 and 16 g/dL, in women, are considered an expression of normality [17]. During pregnancy and/or menstruation, the values are lower due to haemodilution or blood losses. When the need for iron in the body is not satisfied, it is advisable to resort to supplements to treat or prevent iron deficiency or simply to compensate for its deficiency. Dietary supplements are widely used and offer the potential to improve health if appropriately targeted to those in need. Inadequate nutrition and micronutrient deficiencies are prevalent conditions that adversely affect global health. Although improvements in diet quality are essential to address these issues, dietary supplements or food fortification could help individuals at risk of deficiencies meet the requirements [18,19,20]. It is noteworthy that iron supplements can also cause side effects or imbalances in the levels of other essential nutrients, especially metals such as copper and zinc [21]. These contraindications can lead the individual to intolerance of the iron supplement, or even to the possible rejection of it. It is therefore necessary to evaluate the quality of the supplement. For this reason, there are many iron formulations alternatives to prevent or treat iron deficiency anaemia. Organic formulations such as iron gluconate, iron pyrophosphate, iron bis-glycinate, etc. have components that reduce side effects and improve solubility. However, it was found that these formulations did not always have an immediate effect on the individual and were therefore not often used to manage severe iron deficiency [22].

In the past, to compensate for iron deficiency, a popular method involved the use of an apple “contaminated” with iron. The method consisted of inserting iron nails into an apple for a night, in order to transfer a small part of the iron to the apple, because organic acids naturally contained in the apples (alpha-hydroxy acids, such as malic acid, tartaric acid, citric acid etc.) made the nails rust and the iron thus yielded was absorbed by the pulp of the fruit in the form of ferrous organic complexes (among which is the citrate iron (II) complex). The following day, the apple with the composite extracted iron from the nail specimen was ingested by the patient. In the literature, there is a patent on the preparation of iron citrate [23], which has never been commercialized due to the high costs and complex procedures of the preparation of the compound. The hydrothermal synthesis of coordination polymer of iron (II) citrate was described by Birsa Čelič et al. [24] as potential candidates for biomedical applications. Starting from these premises, the current study evaluated the production and characterization of an iron (II) citrate complex by direct reaction between iron filings and citric acid in aqueous solution. 

## 2. Materials and Methods 

### 2.1. General Experimental Information 

All reagents, solvents and standards used to produce the iron citrate neutral complex were obtained from different suppliers and all were of high purity: iron filings (>99%), citric acid monohydrate (>99.5%), iron sulphate heptahydrate, 1,10-ortho-phenanthroline and potassium thiocyanate pure for analysis were purchased from Sigma-Aldrich (Buchs, Switzerland). Ultrapure water was produced by a milli-Q generator (Millipore, Bedford, MA, USA). Potassium sulfocyanide pure for analysis from Carlo Erba (Milan, Italy). Analytical balance series B 204 S (Mettler-Toledo, Germany), freeze dryer mod. Heto Lyolab 3000 (Analytical De Mori, Milan, Italy). UV-Vis spectrophotometer, mod UV-1601 (Shimadzu, Tokyo, Japan), quartz cuvettes with optical path of 1.00 cm. Automatic powder diffractometer Panalytical Empyrean Powder (Santa Barbara, CA, USA) and automatic analyser for the determination of total organic carbon Skalar Analytical B.V (Breda, The Netherlands) were used. Thermogravimetric analyser TGA400 Perkin Elmer (Norwalk, CT, USA).

### 2.2. Synthesis of Iron Citrate Complex

Citric acid monohydrate (21 g) were added to 500 mL of ultrapure water and the solution was heated under magnetic stirring. The iron filings (5.5 g) were added when the temperature of the solution reached 90 °C and the mixture was cooled at room temperature after the complete reaction of the iron. The precipitate grey/pearly salt was washed with water and filtered off under a vacuum with a paper filter; finally, the residue was freeze-dried after freezing at −20 °C. 

### 2.3. Thermogravimetric Analysis (TGA)

Thermogravimetric analysis (TGA) was conducted for iron citrate (~100 mg) in a temperature range from 30 °C to 900 °C. Data are shown as the average of three independent replicates. 

### 2.4. Determination of Iron Oxidation State

The colorimetric method based on the complex ion formed with three molecules of 1,10-phenanthroline and one of iron (II) ion was used to detect the oxidation state of iron (II) ion [25]. 

The complexation of the iron (III) ion with thiocyanate (SCN^−^) has been useful to detect the oxidation state of iron (III) ion as reported by Lister et al. [26]. In this case, the sensitivity of these colorimetric methods (0.1–0.5 ppm) was sufficient for our purposes.

The eventual colour (red) appearing in the solution, during the first or the second mentioned assays, indicates the presence of iron (II) or iron (III) ions, respectively. 

Spectrophotometric analyses were performed, for quantitative analyses, at the wavelength of 520 nm and 480 nm, that are maximum absorption values of 1,10-phenanthroline and thiocyanate complexes, respectively.

For the spectrophotometric assay of iron, the required solutions were prepared from analytical grade ammonium iron (II) sulphate [(NH_4_)_2_Fe(SO_4_)_2_×6H_2_O], 1,10-phenanthroline, potassium thiocyanate (KSCN), hydroxylamine hydrochloride (NH_2_OH×HCl), sodium acetate (NaAc) and 99.999% H_2_SO_4_ (Aldrich product # 339741).

### 2.5. Complex Stability 

Iron citrate (50 mg) was added to 100 mL of ultrapure water. The pH value (6.5) of the solution was modified using NaOH (0.1 M) or HCl (0.1 M). The evaluated pH range was from 2 to 12. The stability of the complex was evaluated via the spectrophotometric techniques described in Section 2.4.

### 2.6. X-ray Diffractometry 

The X-ray diffraction profiles of the synthesized samples were recorded with an automatic powder diffractometer, using the Kα radiation of Cu filtered with Ni, which corresponds to a wavelength of 1.5418 Å. The profiles were recorded using a continuous scan in the range of the 2θ diffraction angle between 5 and 60°.

### 2.7. Attenuated Total Reflectance (ATR) Analysis 

A weighted amount of iron citrate was set in a plate of zinc selenide (ZnSe) and the depth of penetration in the sample of the incident ray was about 0.5 μm. The spectra were acquired between 4000 cm^−1^ and 700 cm^−1^ and reported in absorbance units, with the last spectrum recorded during cleaning serving as the reference. Where needed, signals from the water gas-phase spectrum in the 4000–700 cm^−1^ range were subtracted.

### 2.8. Total Organic Carbon Analysis (TOC)

Iron (II) citrate (70 mg) in solid form was subjected to elemental carbon analysis in order to evaluate the percentage of carbon present in it. Samples were oxidized with phosphoric acid and ammonium persulphate in the reaction chamber and then gaseous flow was divided into two parts in order to test the amount of organic carbon and inorganic carbon. Hence, one of them was used to evaluate the inorganic carbon obtained after oxidation via the thermal combustion method. The second part was used to test the organic carbon and was bombarded with UV ray emitted by a mercury vapor lamp. In this way, free radicals react with carbon to produce CO_2_. Both sample parts were passed to the membranes to measure their conductivity via an automatic analyser for the determination of total organic carbon.

## 3. Results and Discussion

The iron (II) citrate complex was obtained by direct reaction from the reactants as excess citric acid and iron filings, exploiting a hot oxidation reaction. The synthesis reactions were carried out by maintaining an excess of citric acid in the reaction environment in such a way as to guarantee the complete transformation of the introduced iron. The iron has been used in the form of filings in order to have a higher reaction surface for the iron itself. As reported in Section 2, when the solution of citric acid in water was almost boiling, the iron filings were added. The redox reaction of iron with citric acid in the presence of water led to the formation of the iron citrate with a stoichiometric 1:1 reaction, in which Fe was coordinated with two oxygen atoms, then releasing hydrogen in the gaseous form. The slow in situ generation of iron (II) favours the formation of a crystalline solid keeping oversaturation to low levels. Furthermore, the reducing environment avoided the formation of Fe (III).

The reaction is as follows:

Fe (s) + C_6_H_8_O_7_ (s) = FeC_6_H_6_O_7_ + H_2_ (g). (1)

Based on the stoichiometric ratio 1:1 of the reagents (citric acid and iron filings), the iron theoretical percentage is 22.7% FeC_6_H_6_O_7_ and 21.2% (FeC_6_H_6_O_7_∙H_2_O).

(2) $$\%\mathrm{Fe}({\mathrm{FeC}}_{6}H_{6}O_{7})=\frac{\mathrm{AW}\left(\mathrm{Fe}\right)}{\mathrm{MW}\left({\mathrm{FeC}}_{6}H_{6}O_{7}\right)}\times 100=\frac{55.845}{245.95}\times 100=22.7\%$$

(3) $$\%\mathrm{Fe}({\mathrm{FeC}}_{6}H_{6}O_{7}\cdot H_{2}O)=\frac{\mathrm{AW}\left(\mathrm{Fe}\right)}{\mathrm{MW}\left({\mathrm{FeC}}_{6}H_{6}O_{7}\cdot H2O\right)}\times 100=\frac{55.845}{263.963}\times 100=21.2\%$$

**Figure 1:**
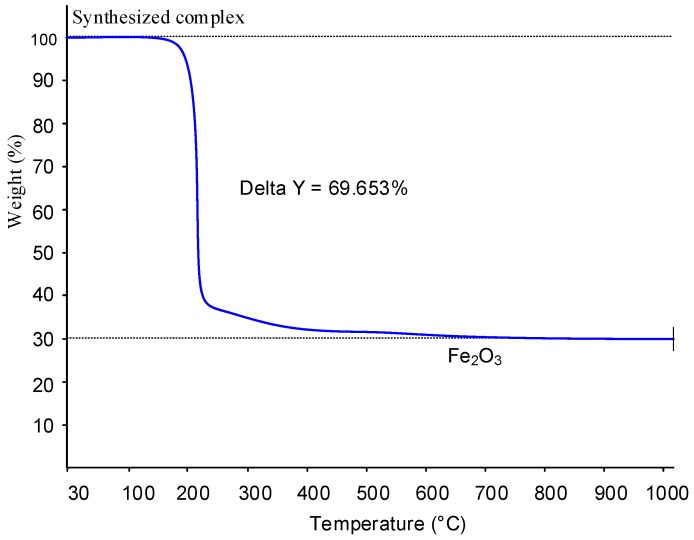
TGA analysis of iron citrate powder. The temperature increased from 30 °C to 900 °C at 200 °C min^−1^.

Thermogravimetry led to the easy determination of the percentage by weight of iron in the iron citrate powder. In fact, from the data obtained, the percentage of iron in the samples was 21.1% ± 0.4 (*w*/*w*). Hence, the results are in good agreement with the theoretical value calculated for (FeC_6_H_6_O_7_∙H_2_O) (Figure 1).

Total carbon analysis of the solid iron citrate complex was also performed. Based on the stoichiometric ratio 1:1 of the components, the carbon theoretical percentage is 29.3% (FeC_6_H_6_O_7_) and 27.3% (FeC_6_H_6_O_7_∙H_2_O).

(4) $$\%C({\mathrm{FeC}}_{6}H_{6}O_{7})=\frac{\mathrm{AW}\left(C\right)\times 6}{\mathrm{MW}\left({\mathrm{FeC}}_{6}H_{6}O_{7}\right)}\times 100=\frac{72.066}{245.95}\times 100=29.3\%$$

(5) $$\%C({\mathrm{FeC}}_{6}H_{6}O_{7}\cdot H_{2}O)=\frac{\mathrm{AW}\left(C\right)\times 6}{\mathrm{MW}\left({\mathrm{FeC}}_{6}H_{6}O_{7}\cdot H2O\right)}\times 100=\frac{72.066}{263.963}\times 100=27.3\%$$

Collected data are in good agreement with the theoretical value for FeC_6_H_6_O_7_∙H_2_O. In fact, the total carbon percentage in the iron citrate samples is 27.2 ± 0.1% (*w*/*w*) (average of three samples).

In light of the total carbon analysis, the thermogravimetric data in Figure 1 can be interpreted in an alternative way to prove that the content of hydrogen and oxygen of the iron citrate complex is consistent with the elemental composition FeC_6_H_6_O_7_∙H_2_O = FeC_6_H_8_O_8_.

In fact, since during the TGA analysis, the iron citrate complex is converted to Fe_2_O_3_(s), the weight loss of 69.653% exposed in Figure 1 implies that 69.653 g of carbon, hydrogen and oxygen are lost per 100 g of the iron complex.

Now, if the atomic composition of the iron complex, with respect to C, H and O, is C_x_H_y_O_w_, the 69.653 g per 100 g of the complex that is lost during the TGA analysis corresponds to the elemental composition C_x_H_y_O_w−1.5_ (because not all oxygen is lost and 1.5 atoms of oxygen per mole of the iron complex remain in the Fe_2_O_3_ (s) product).

After the carbon analysis, we know that 27.2 ± 0.1 g of carbon are obtained per 100 g of the solid iron citrate complex. In consequence, the amount of (H_y_O_w−1.5_) from the array of atoms C_x_H_y_O_w−1.5_ that is lost per 100 g of iron citrate complex is experimentally determined to be (H_y_O_w−1.5_) = 69.653−27.2 = 42.5 ± 0.1 g. 

If we assume that the solid iron citrate complex is FeC_6_H_6_O_7_, then (H_y_O_w−1.5_) = (H_6_O_5.5_). Under this assumption, only 38.2 g of (H_6_O_5.5_) would be obtained from 100 g of FeC_6_H_6_O_7_.

On the contrary, if we assume that the solid iron complex is FeC_6_H_6_O_7_∙H_2_O = FeC_6_H_8_O_8_, then (H_y_O_w−1.5_) = (H_8_O_6.5_) and, from 100 g of FeC_6_H_6_O_7_∙H_2_O, would theoretically be obtained 42.43 g of (H_8_O_6_). This theoretical value (42.43 g) agrees very well with the value found by combining the TGA and total carbon analysis (42.5 ± 0.1 g) and confirms the composition FeC_6_H_6_O_7_∙H_2_O for the iron citrate complex (as would the elemental analysis of hydrogen and oxygen in the solid).

The spectrophotometric method, based on the complex formed by iron (II) with 1,10-phenanthroline (Phen), i.e., Fe^2+^ + 3Phen ⇌ Fe(Phen)_3_^2+^, has been employed both for the analysis of total iron and iron (II) amounts in the iron citrate complex, using two slightly different procedures that are described below.

A 1g/L iron (II) standard solution was prepared by dissolving 7.0226 g of ultrapure (NH_4_)_2_Fe(SO_4_)_2_×6H_2_O (MW = 392.14 u) in deaerated water up to the volume of a 1L volumetric flask. From this, a secondary standard, 100 mg/L of iron (II), was prepared by diluting 1:10 with deaerated water. 

Ten calibrating solutions, of known iron (II) concentration, ranging from 0.2 to 2.0 mg/L (or, 3.58×10^−5^ M to 3.58×10^−6^ M), were prepared by mixing, in 100-mL volumetric flasks, measured volumes of the 100 mg/L Fe(II) standard solution with fixed volumes of 2 M H_2_SO_4_, 10% *w*/*w* NH_2_OH×HCl, 0.25% *w*/*w* 1, 10-phenanthroline (Phen) and 2.0 M sodium acetate, according to Table 1.

The pH of the calibrating solutions was 4.0 ± 0.2. Addition of an excess of hydroxylamine hydrochloride, which reduces iron (III) to iron (II) (2NH_2_OH + 4Fe^3+^ →N_2_O + 4Fe^2+^ + H_2_O + 4H^+^), keeps iron completely in the +2 oxidation state.

The spectrophotometer was calibrated by measuring the absorbance at 520 nm of the calibration solutions in Table 1, using 1.00 cm quartz cuvettes. Absorbance was measured against a reagent blank that was prepared in the same way as the calibration solutions, except that the 100 mg/L standard solution was not added.

The experimental data points (Abs., conc.) were well interpolated by a least squares regression line with a coefficient of determination *R*^2^ = 0.9993 (Abs. = Absorbance at 520 nM; Conc. = iron(II) concentration, mg/L).

Then, to the experimental (Abs., conc.) data, the MS Excel statistical function LINEST was applied, which is a matrix function that calculates several statistical parameters from an array of (*y*, *x*) data points. 

From collected (Abs., conc.) data, the following statistics were evaluated: the slope (*b* = 0.191 L/mg) and intercept on the absorbance axis (*a* = 0.0097) of the least square regression line (*y* = *a* + *bx*) through the experimental points; the standard deviations of slope (s_b_ = 0.0017 L/mg) and of intercept (s_a_ = 0.0021); and, finally, the standard deviation of experimental points around the regression line (s_y/x_ = 0.0030).

From the standard deviation of intercept (s_a_) and the slope (*b*) of the regression line, the limit of detection (LOD) of the method is evaluated to be 0.036 mg/L (i.e., LOD = 3.3 × s_a_/*b* mg/L) [27].

The standard deviation around the regression line (s_y/x_) is used to evaluate the standard deviation of iron concentrations by interpolating the calibration curve with the measured absorbance of samples.

Iron, in the iron citrate complex, was determined by weighing 0.2500 ± 0.0002 g of the solid sample directly in a 250 mL volumetric flask. The solid sample was then completely dissolved by adding 2 M H_2_SO_4_ up to the volume of the volumetric flask. Before use, a stream of nitrogen was passed through the 2 M H_2_SO_4_ solution for several hours in order to expel the oxygen, which would oxidize iron (II) to iron (III). After preparation, much care was taken to avoid oxygen absorption by the solution and to preserve the original oxidation state of iron in the dissolved solid.

For this solution, the total iron concentration was determined very simply, by transferring 0.5 mL of the solution to a 100-mL volumetric flask and adding to it the same volume of reagents used during the preparation of the calibrating solutions (see Table 1). However, to keep the pH of the measured sample solution within the range of the calibration standards (4 ± 0.2), the volume of 2 M H_2_SO_4_ solution added was reduced to 1.5 mL.

These operations result in the determination, from the measured absorbance, of the total iron concentration in the above iron citrate complex solution. In fact, even if iron (III) is present in the sample, it will be reduced to iron (II) by hydroxylamine. 

This experiment was repeated three times and the three values of the total iron concentration were averaged. To the average value of total iron concentration, a 95% confidence level interval was attached, as evaluated from the standard deviation around the regression (s_y/x_) [27]. 

The total iron concentration in the sample solution, evaluated assuming for the iron citrate complex the composition FeC_6_H_6_O_7_∙H_2_O, was 1.058 ± 0.010 mg/L, and compares very well with the average concentration, 1.082 ± 0.025 mg/L, determined from the three replicates of the analysis. 

Exactly the same experiment was repeated with a second 0.5-mL portion of the iron citrate complex solution, except that, in this case, the hydroxylamine solution was substituted with a sodium fluoride (NaF) solution, which masks iron (III). Furthermore, before use, oxygen was expelled from all the added solutions by purging with a nitrogen flux and the prepared solution, carefully kept out of contact with the atmosphere, was, after a very short delay, presented to the spectrophotometer and its absorbance measured. In this way, only iron (II) was determined from the measured absorbance. 

As in the previous case, this experiment was repeated three times and three values of the iron (II) concentration were obtained and averaged.

Obviously, if the dissolved iron citrate complex contains a significant fraction of iron in the form of iron (III), the total iron concentration will be greater than the iron (II) concentration and from the difference (total iron concentration – iron (II) concentration), it is possible to evaluate the amount of iron present as iron (III). 

However, the average of the three iron (II) concentrations, determined by the above procedure, was 1.070 ± 0.025 mg/L and cannot, within the uncertainty limits, be distinguished from the total iron concentration. So much so that we can safely conclude that, in the iron citrate complex, iron is practically completely present as iron (II) and that the above spectrophotometric assays of iron support the composition FeC_6_H_6_O_7_∙H_2_O for the analysed iron citrate complex (since both the total iron concentration and the iron (II) concentration are, within their uncertainty, identical with the expected iron concentration). 

The above solution of the iron complex was also tested for iron (III) using the thiocyanate method. In fact, thiocyanate is generally used for the detection and assay of iron (III) because of the formation of red iron (III) thiocyanate complexes, which absorb strongly at 480 nm. However, in agreement with the above results, no measurable absorption could be detected when samples of the solution of the iron citrate complex were treated, under controlled conditions, with a deaerated 15% *w*/*w* potassium thiocyanate solution.

Finally, the collected data show the exclusive presence of iron (II) ion in the synthesized iron citrate complex and this represents an advantage over other formulations of supplements that contain iron (III) ion. In fact, iron (II) is better assimilated (high bioavailability) compared to iron (III) [28].

For its use as an iron supplement, it is necessary to evaluate the stability at different pH of iron citrate complexes. For example, inside the human stomach the synthetic product is exposed at a very low pH and an evaluation of the stability at different pH allows one to understand whether the supplement could be taken as such or if it would be preferable to use a protective film for the stomach that would allow the dissolution and assimilation of iron in the intestine. 

In this respect, spectrophotometric assays to determine the oxidation state of iron reported above were conducted for iron citrate solutions of different pH (from 2 to 12), confirming the stability of iron ion in the oxidation state +2 in the iron citrate complex. Furthermore, there was no precipitation of iron as ferrous hydroxide or ferric hydroxide in the evaluated pH range. 

**Figure 2:**
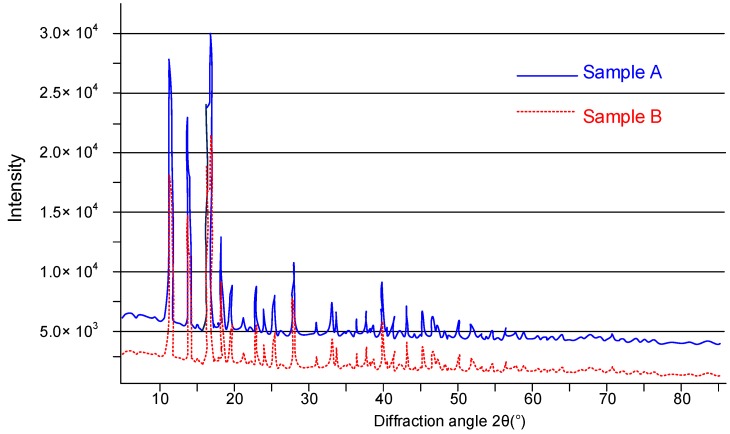
Comparison between X-ray diffraction spectra of two samples of iron citrate powder. Samples A and B are two independent replicates.

Two samples of iron citrate powder were subjected to X-ray analysis, obtaining a series of spectra that showed the presence of a crystalline-type material. Figure 2 shows the spectra, which confirm that the synthesis procedure allowed us to obtain a crystalline compound. Finally, the sample obtained in crystalline form was subjected to a re-crystallization test to try to obtain single crystals. However, until now, it has not been possible to obtain a well-defined single crystal.

**Figure 3:**
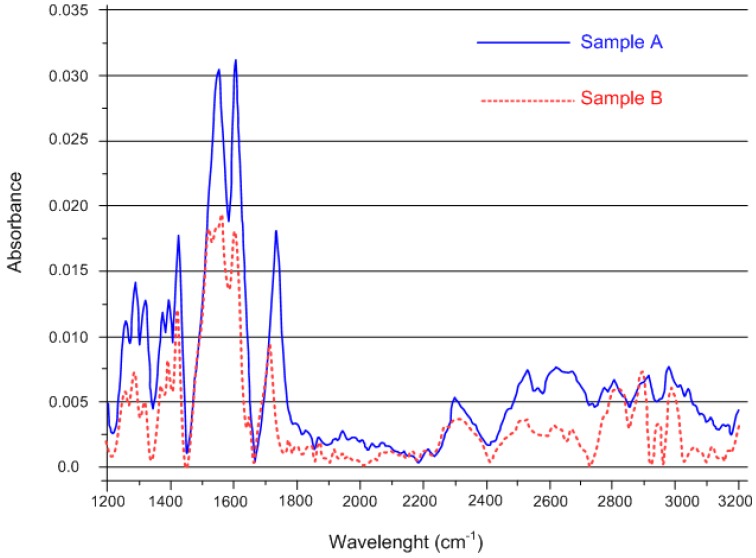
Comparison between IR spectra of two samples of iron citrate powder. Samples A and B are two independent replicates.

In order to verify the hypothetical coordination by the metal with the organic citrate type sites, an infrared spectroscopy (IR) analysis of a solid sample of citric acid and sodium citrate was carried out, stratifying the samples on the cell using ethanol. The ATR spectra of the samples of iron citrate were compared with those of citric acid and sodium citrate. Particularly interesting was the comparison of the spectra obtained for two samples of iron citrate powder, in particular from the peaks relative to the zone of the signals related to the COO^−^ showing a typical band around 1600/1700 cm^−1^. The presence of metal-related peaks, which were found only for the samples of iron citrate (Figure 3), allowed us to establish the right coordination linking by the citrate with the iron.

Birsa Čelič et al. [24] previously reported the synthesis of a neutral carboxylated ferrous citrate compound that is similar to the iron (II) citrate complex that is presented in the current study. However, the carboxylated ferrous citrate polymer was hydrothermally synthesized by reacting ferrous chloride with citric acid in a basic solution. Consequently, ionized citric acid in solution is carboxylated by irregular bonds to iron (II), in which iron (II) is octahedrally complexed by different oxygen atoms. Complexation of [Fe(H_2_cit)(H_2_O)]n times, and the crosslinking between iron (II) citric acid, resulted in the formation of an iron polymer compound. As confirmed by single-crystal X-ray diffraction, the structure of the compound can be described as a pseudo-three-dimensional framework composed of infinite chains of coordinated polyhedral iron. Indeed, the iron atoms are hexacoordinated by two partially deprotonated citrates and a water molecule in a distorted octahedral fashion. Furthermore, the reaction was carried out in an autoclave at a temperature of 150 °C (423 K) for one day and the polymer maintains its magnetic proprieties and stability at temperatures up to 275 °C (548 K). In contrast, an iron (II) citrate complex was formed by the reaction of iron filings and citric acid in 2 h in the current study. The diffraction spectra obtained from the two compounds cannot be superimposed, especially in the region between 40 and 45° (diffraction angle 2θ), thus indicating that the two compounds are different. The ferrous citrate compound reported in the literature showed the coordination of iron (II) in a complex octahedral structure, possibly as a long chain of polymers rather than a 1:1 coordination of iron ions and dibasic citrate in our compound, which was formed by the dehydrogenation of citrate with consequent formation of a neutral complex (see the reaction (1)). Finally, and most importantly, the compound that was reported earlier focused on the preparation of iron citrate metal complex with magnetic properties that could be potential candidates for biomedical applications rather than the synthesis of an iron compound that is to be used as a food supplement or an additive for the management of iron deficiency.

The good results obtained from the chemical characterization of the iron citrate complex led us to consider its potential application as an iron supplement. In fact, the enrichment of foods with iron citrate would allow individuals with iron deficiency to not change their eating habits and at the same time avoid taking pharmaceutical formulations to supplement this element in the body.

## 4. Conclusions

The current study shows a rapid and easy method to synthesise iron (II) citrate complex from iron filings and citric acid. The complex was subjected to a battery of analytical techniques to demonstrate that the synthesised crystalline solid corresponds to the formula FeC_6_H_6_O_7_∙H_2_O. The relevance of the synthesised complex, exclusively containing iron in the +2 oxidation state, is associated with its potential use as an alternative to commercially available iron supplements and additives. 

Further studies, some already in progress, will be conducted on the synthesised complex in order to obtain data with undoubted value for the realization of a food supplement. In this respect, further research is needed to validate the real effectiveness and mechanism of action of this complex. In vitro and in vivo studies will be performed, first on cells and animal models, in order to evaluate its safety and toxicity. Finally, the assimilation within the human body will be evaluated in human volunteers. Furthermore, enrichment tests will be performed on various food matrices to evaluate the macroscopic effects of the iron (II) citrate complex on the enriched foods.

## Figures and Tables

**Table 1:** Preparation of iron (II) calibrating solutions.

Standard Fe (II) 100 mg/L	H_2_SO_4_ 2M	NH_2_OH × HCl 10% *w*/*w*	Phen 0.25% *w*/*w*	NaAc 2 M	Total Volume (with H_2_O)	Conc. Fe (II)
mL	mL	mL	mL	mL	mL	mg/L
0.200–2.00	2.00	1.00	5.00	5.00	100	0.200–2.000
